# Mental distress and associated factors among undergraduate students at the University of Hargeisa, Somaliland: a cross-sectional study

**DOI:** 10.1186/s13033-017-0146-2

**Published:** 2017-06-08

**Authors:** Liban Hersi, Kenfe Tesfay, Hailay Gesesew, Wolfgang Krahl, Deria Ereg, Markos Tesfaye

**Affiliations:** 1 Faculty of Medicine, University of Hargeisa, Hargeisa, Somaliland; 2 Department of Psychiatry Nursing, Mekelle University, Mekelle, Ethiopia; 3 Department of Epidemiology, Jimma University, Jimma, Ethiopia; 4 Discipline of Public Health, Flinders University, Adelaide, Australia; 5 Department of Forensic Psychiatry, Isar Amper Klinikum, Munich, Germany; 6 Department of Psychiatry, College of Health Sciences, Jimma University, Jimma, Ethiopia; 7 Center for International Health, Ludwig Maximillians University, Munich, Germany

**Keywords:** Mental distress, Undergraduate students, Self-reporting questionnaire, Low-income country, Somaliland

## Abstract

**Background:**

Mental distress is a common finding among University students. Empirical research has confirmed that the University student population has a higher prevalence of mental disorder than the general population. However, no previous study has examined the mental health conditions of students in Somaliland.

**Methods:**

An institution based cross-sectional study was conducted on a sample of 570 undergraduate students at the University of Hargeisa in October, 2013. Study subjects were selected using a stratified random sampling. The Self-reporting questionnaire (SRQ-20) was used to assess mental distress. Multiple logistic regression analysis was carried out to identify factors independently associated with mental distress.

**Results:**

The point prevalence of mental distress was found to be 19.8%. Mental distress was associated with being female (AOR = 3.52, 95% CI 1.94, 6.39), having a monthly income of 100 United States dollars (USD) or less (AOR = 2.19, 95% CI 1.12, 4.28), and not having a satisfying relationship with the family (AOR = 11.52, 95% CI 3.18, 41.72) and friends (AOR = 7.33, 95% CI 2.83, 18.93). Nearly one in five students (18.6%) has been using Khat in the previous 12 months. Khat use was also associated with greater likelihood of mental distress (AOR = 2.87, 95% CI 1.26, 6.56). In addition, financial difficulties and the poor prospect of finding a job were common sources of stress among the students.

**Conclusions:**

A significant proportion of the students at the University of Hargeisa suffer from mental distress which might have a detrimental effect on their academic performance. The mental health needs of the University students require attention with special emphasis on female students, students experiencing financial hardships, students who use Khat and those who have interpersonal problems.

## Background

Mental health, defined as “the successful performance of mental functions in terms of thought, mood, and behavior that results in productive activities, fulfilling relationships with others, and the ability to adapt, change, and cope with adversity” [1], is a crucial aspect of overall health for students. Empirical findings have indicated that student populations suffer from higher prevalence of mental disorders than the general population [2]. University students represent a specific population with concerns, burdens and worries that differ from other age and occupational groups. The experiences of students, although often exciting, invigorating and empowering, can also be stressful and may trigger various forms of psychopathology [3].

A number of studies from sub-Saharan Africa have found that a significant proportion of the population suffer from mental distress. Studies examining the prevalence of mental distress in Ethiopia—using varying cut-offs of the Self-reporting questionnaire (SRQ-20) have reported prevalence rates ranging from 11.7 to 25.8% in the community settings [4–6] and from 21.6 to 49.1% among University students [7–11]; and a recent study done in a rural community in Ethiopia has reported the prevalence of psychological distress to be 27.9% [12]. A study from Zambia reported mental distress to be more common among women (15.4%) than in men (12.4%) [13]. Additionally, a study from South Sudan has reported a high prevalence rate of mental distress i.e. 23.2% [14].

Mental health conditions can have profound impact on University students’ functioning. At the individual level, they can affect all aspects of physical, emotional, cognitive and interpersonal functioning. They may also have a negative impact on the academic performance [15]. Students with higher levels of mental distress have higher test anxiety and lower self-efficacy [3]. Furthermore, students with poor mental health influence many other people on campuses, including roommates, classmates, faculty members and staff [3]. A national mental health survey in Australia has reported at least 14% of adolescents younger than 18 years old have experienced a mental or substance use disorder in the preceding 12 months, and the prevalence was nearly double for those between the ages of 18 and 24 years [16]. Studies on the burden of mental disorders indicate that there is substantial variation across nations [17]. Some cultural factors such as, parental involvement in young people’s decision-making and the tendency to form friendships within one’s cultural group might be protective [18]. On the other hand, cultural factors such as, restricted autonomy for women in decision making may have the opposite effect [19].

This study was conducted among University students in Somaliland. Somaliland is an unrecognized self-declared de facto sovereign state that is internationally considered an autonomous region of Somalia. The government of Somaliland regards itself as the successor state to the British Somaliland protectorate, which became independent on June 26, 1960 as the State of Somaliland before uniting with the Trust Territory of Somalia (the former Italian Somaliland) on July 1, 1960 to form the Somali Republic. Somaliland is bordered by Ethiopia in the south and west, Djibouti in the northwest, the Gulf of Aden in the north, and the autonomous Puntland region of Somalia to the east. After the collapse of the central government in 1991, the local government, led by the Somali National Movement, declared independence from the rest of Somalia on May 18th of the same year. Since then, the territory has been governed by an administration that seeks self-determination as the Republic of Somaliland. The local government maintains informal ties with some foreign governments, who have sent delegations to Hargeisa. However, Somaliland’s self-proclaimed independence remains unrecognized by any country or international organization. Since Somaliland is unrecognized, international aid donors have found it difficult to provide aid. As a result, the government relies mainly upon tax receipts and remittances from the large Somalilanders in the diaspora who contribute immensely to Somaliland’s economy. These factors affect student life in that University students do not have access to international academic scholarships and the support from the government of Somaliland for university studies is minimal.

A recent review has highlighted the importance of empowering an environment that ensures the needs of adolescent youth are met so that the healthy future development of communities and countries can be enhanced [20]. The unique challenges faced by the students in Somaliland who try to improve their life situation through tertiary education could put them at high risk of developing mental distress. Poor mental health can interfere with day to day functioning including academic success. Data on the factors associated with mental distress among Hargeisa University students will help in planning interventions to improve their mental health and hence, the academic successes of one of the most vulnerable segments of the community. Therefore, we aimed to investigate the prevalence of mental distress and to identify independently associated factors among undergraduate students in University of Hargeisa.

## Methods

### Study design and setting

A cross-sectional study was conducted among undergraduate students at Hargeisa University, in October 2013. The University of Hargeisa is one of the first public higher educational institutions in Somaliland that was established by the government of Somaliland in 1999. The University has nine faculties offering bachelor degrees and one postgraduate diploma on peace and development studies. The number of students enrolled at the University was approximately 6000. Students are not provided with dormitories. Students or their relatives have to pay all expenses including tuition fees and living expenses. While Somali is the mother tongue for the majority of the students, all are proficient in English since this is the language for academics. Khat use (a shrub which contains amphetamine like substance called cathinone) is legal and is widely consumed by the community while alcohol is illegal and rarely consumed in Somaliland.

### Participants and sampling

The sample size was calculated using single population proportion formula. The prevalence of mental distress was assumed to be 50% to achieve maximum sample size. Six hundred students were selected using stratified sampling technique from all undergraduate programs. The sample was allocated proportionally to each of the departments. Systematic random sampling was used to recruit students from each department. Students who were on sick leave during the data collection period were excluded.

### Instruments and data collection

Data on socio-demographic and socio-economic characteristics were collected using a structured pre-tested questionnaire. Data regarding the monthly income included income from a range of sources including financial support from relatives, friends, and income from part-time employment. The English version of the World Health Organization’s SRQ-20 was used to assess for mental distress [21]. The English version was chosen because the study participants were proficient in English and the SRQ-20 was not validated in the Somali language. The SRQ-20 consists of 20 questions asking whether the respondent experienced symptoms of anxiety, depression, or somatic symptoms such as headache during the preceding 30 days. The SRQ-20 was developed for international use and was selected because of a better face validity than other tools. In addition, SRQ-20 has been widely used as a measure of mental distress among University students in neighboring Ethiopia [4–9, 11]. There is no established cut-off for the Somali population. A study done in different low income countries has used varying cut-offs ranging from 5 to 11, and found sensitivity of 73–83% and specificity of 72–85% [22]. Additionally, an open ended question: “What are the most important stressors for you at present?” was included and respondents were requested to elaborate their answers. Data was collected through self-administered paper-based questionnaires distributed to selected participants by two nurses from Hargeisa hospital and two medical students from Hargeisa University who were trained for 1 day on the data collection procedures. A medical doctor and the principal investigator supervised the data collection.

### Data analysis

Data exploration and cleaning were conducted before analysis. The analysis of both descriptive and inferential statistics was performed. Descriptive statistics included mean and standard deviation for continuous data, and percentage and frequency tables for categorical data. Mental distress was defined as having 11 or more symptoms on the SRQ-20. Bivariate logistic regression was done to explore for the factors associated with mental distress and select variables for the multivariable logistic regression. Variables with *p* value of <0.25 on bivariate analysis were entered into multiple logistic regression. On multiple logistic regression, variables with p value <0.05 were considered as factors independently associated with mental distress. The data were summarized using odds ratio (OR) and 95% confidence interval. The analyses were conducted in Statistical Package for the Social Sciences version 16. The open-ended question regarding the sources of stress was analyzed by listing the responses and grouping them thematically. The most frequent themes were presented along with explanatory statements in the respondents’ own words.

## Results

A total of 570 students participated in the study representing a response rate of 95%. The majority (60.7%) of the participants were male. The mean age (standard deviation) of the participants was 23.5 (4.4) years. Being single marital status was over represented (87.9%), and over half (51.2%) received financial support from their parents. About three-fourth of the students (72.0%) had monthly income of 100 United States dollars (USD) or less (Table 1).

**Table 1:** Background characteristics of study participants from University of Hargeisa (n = 570)

Characteristics	Number	Percentage
Age in years		
Mean ± SD	23.5 ± 4.4	
Sex		
Male	346	60.7
Female	224	39.3
Level of education (year)		
1st	137	24.0
2nd	140	24.6
3rd	137	24.0
≥4th	156	27.4
Marital status		
Single	501	87.9
Married	57	10.0
Other	12	2.1
Monthly income ($)		
≤100	308	72.0
>100	120	28.0
Main source of financial support		
Parent	292	51.2
Self	107	18.8
Siblings	72	12.6
Relative	72	12.6
Others	27	4.7
Part-time job		
Yes	204	35.8
No	366	64.2
Family in Hargeisa		
Yes	467	81.9
No	103	18.1
Field of study		
Science & technology	115	20.2
Business	107	18.8
ICT	93	16.3
Economics	81	14.2
Law	36	6.3
Arabic and Islamic	36	6.3
Medicine	34	6.0
Engineering	40	7.0
Education	28	4.9

**Fig. 1:**
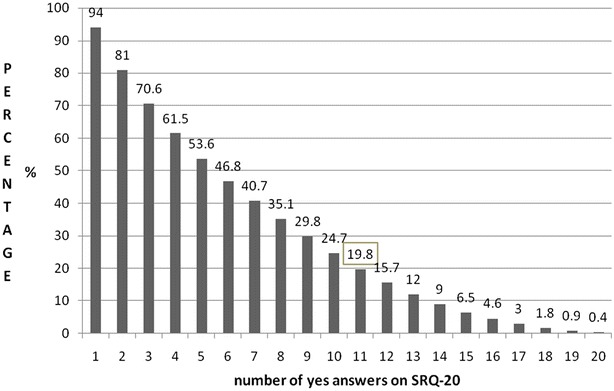
Percentage distribution of mental distress for the different cut-off points on the SRQ-20

The number of symptoms of mental distress on SRQ-20 ranged between 0 and 20. The mean score (standard deviation) for SRQ-20 scores was 6.4 (4.6). The overall point prevalence of mental distress was 19.8% (95% CI 16.6–22.9%) (Fig. 1). The 12 month prevalence of Khat use, smoking and drinking were 18.6, 9.1 and 1.8%, respectively.

After adjusting for confounders, being female, lower monthly income, not having close friends, a non-satisfying relationship with friends or families, and use of Khat were associated with mental distress. Female students were 3.5 times more likely to have mental distress than male students (AOR = 3.52, 95% CI 1.94, 6.39). Students who had monthly income of 100 USD or less were 2.2 times more likely to have mental distress than those who had monthly income >100 USD (AOR = 2.19, 95% CI 1.12, 4.28). Open-ended questions revealed that financial difficulties and difficulty finding jobs were the most common stressors that students reported. For example, one student stated that ‘*financial problems and finding a sustained job’* as one of his main stressors. Students’ financial problems were mostly related to unemployment. Another student stated, *‘I am feeling stressed, every time I am thinking of getting a job’.* Another student revealed, ‘*I am stressed about the future on how to get a job after graduating’*.

Students who had no close friend were 2.3 times more likely to have mental distress than those who had close friends (AOR = 2.30, 95% CI 1.02, 5.21). Not having a satisfying relationship with friends (AOR = 7.33, 95% CI 2.83, 18.93) and family (AOR = 11.52, 95% CI 3.18, 41.72) were associated with greater likelihood of having mental distress (Table 2). Many students mentioned: quarrelsome friendships, preparation for marriage and family problems as frequent sources of stress. Students who used Khat in the previous 12 months were 2.9 times more likely to have mental distress (AOR = 2.87, 95% CI 1.26, 6.56) than those who did not use Khat.

**Table 2:** Multiple logistic regression for factors associated with mental distress among undergraduate students at Hargeisa University (n = 570)

Characteristic	Mental distress^a^		Unadjusted odds ratios (95% CI)	Adjusted odds ratios (95% CI)
	Yes	No		
Sex				
Male	13.6	86.4	1	1
Female	29.5	70.5	2.66 (1.75, 4.05)	3.52 (1.94, 6.39)
Age in years				
18–22	25.5	74.5	2.00 (1.31, 3.05)	1.69 (0.96, 3.00)
>22	14.6	85.4	1	1
Marital status				
Single	20.2	79.8	1	–
Married	17.5	82.5	0.84 (0.41, 1.73)	–
Other	16.7	83.3	0.79 (0.17, 3.67)	–
Monthly income (in US dollars)				
≤100	21.8	78.2	1.99 (1.14, 3.50)	2.19 (1.12, 4.28)
>100	12.2	87.8	1	1
Having a close friend				
Yes	16.8	83.2	1	1
No	40.8	59.2	3.41 (2.01, 5.79)	2.30 (1.02, 5.21)
Satisfying relationship with friends				
Yes	17.9	82.1	1	1
No	48.6	51.4	4.32 (2.15, 8.69)	7.33 (2.83, 18.93)
Satisfying relationship with family				
Yes	18.7	81.3	1	1
No	55.6	44.4	5.45 (2.10, 14.15)	11.52 (3.18, 41.72)
Current use of Khat				
Yes	32.1	67.9	2.30 (1.43, 3.70)	2.87 (1.26, 6.56)
No	17.0	83.0	1	1
Current use of alcohol				
Yes	40.0	60.0	2.76 (0.77, 9.94)	0.26 (0.04, 1.84)
No	19.5	80.5	1	1
Current smoking				
Yes	36.5	63.5	2.60 (1.42, 4.77)	2.14 (0.80, 5.75)
No	18.1	81.9	1	1

^a^The numbers indicate percentages

The other source of stress mentioned by the students was academic related factors such as the burden of the long hours of studying for examinations. Medical students also complained about being stressed by the oral clinical examinations. Also, traveling long distance to the university campus from their homes was mentioned as stressor.

## Discussion

One in five students at University of Hargeisa had mental distress. Female gender, lower monthly income, not having close friendships, and not having satisfying relationship were associated with mental distress. Khat use was also associated with greater likelihood of mental distress. However, alcohol use and smoking were not associated with mental distress.

The prevalence of mental distress in this study was comparable to findings from students in Adama University, 21.6% which used a similar tool and cut-off point [7]. Also, a study from Nepal has reported a similar prevalence of psychological morbidity [23]. On the other hand, the prevalence of mental distress reported in our sample is relatively lower than reported by other studies from Ethiopia [8–11]. However, these studies have used lower cut-off points than that of our study. Also, a high prevalence of mental distress has been reported among medical students in low- and middle-income countries ranging from 41.9% in Malaysia [24] to 47.0% in southern India [25]. It is not surprising that these studies found higher prevalence of mental distress as their samples were selected from medical schools only while our study participants were selected from a wide range of fields of study. Of note the prevalence of mental distress in our study is likely a rather conservative estimate given a higher cut-off point on the SRQ-20. Approximately half of the students in our sample would have been cases of mental distress if a lower cut-off of 5/6 was used (Fig. 1).

Studies from high income countries have reported poor financial status to be associated with poor mental health among University students [26, 27]. The fact that students in Somaliland are expected to cover tuition fees and their own living expenses may add to their mental distress. In support of this, the students have mentioned financial hardships to be a common source of stress. Furthermore, students with financial difficulties may be at risk of dropping out from their studies. Parental support in Somaliland may have played a role to buffer the effects of financial hardships among students at University of Hargeisa. Furthermore, the vast majority of the students had their family, who may provide social and emotional support, living in the same city. Our findings suggest that young people from poor families who live outside of the city of Hargeisa face enormous challenges to their study in the University. In addition, students from poorer families who manage to join the university suffer from poorer mental health. Our findings are consistent with the existing evidence that symptoms of anxiety and depression are generally more prevalent among women than men. A study done among Nigerian University students has found that depression was more prevalent among female University students [28]. Similarly, studies from the Middle East countries have also reported that female University students suffer from greater burden of symptoms of anxiety and depression than male counterparts [29–31]. These findings may be due to differences in the rates of exposure to biological and environmental risk factors. Our observation that female students and students with financial hardships had poorer mental health status suggests the need for further assessment of the role of social determinants of mental health in this population.

Students who did not have close friends and those who did not have satisfying relationships with their friends or relatives had higher prevalence of mental distress. Difficult relationships were also mentioned as sources of stress by the participants strengthening the observed statistical associations. A study from Ethiopia has reported students who had difficulty in making friends and conflict with fellows in dormitories had higher prevalence of mental distress [9]. Another study from the US has reported that the prevalence of depression was higher in absence of strong social support [32]. Therefore, strategies designed to improve the quality of interrelationships among University students might promote their mental well-being.

Khat was a commonly used psychoactive substance among the students. Our findings also indicate that Khat use was associated with mental distress. An association between Khat use and mental distress and Khat use was reported by a community based study in Ethiopia [6]. Other studies have found no such association [7]. Studies of substance use suffer from social desirability bias. Hence, the reported findings are likely to be underestimates. In particular, the prevalence of alcohol use may be due to lack of access and its legal prohibition in Somaliland. Nevertheless, the use of Khat might be a coping strategy for the demands of studying and other stressors. However, this needs further exploration using qualitative methods.

Our findings are limited by the use of a screening tool to assess mental health status. There is no established cut-off for the Somaliland community. We have used a relatively high cut-off to achieve a better positive predictive value [21]. However, this might have underestimated the prevalence of mental distress in our study population. In addition, the absence of prevalence estimates from a community based control group sample limits the interpretation our findings. Causal associations cannot be established due to the cross-sectional study design used. Students with worse financial situations and poorer mental health might have dropped out; therefore, the possibility of selection bias exists. Nevertheless, this is the first study to report the mental health status of University students in Somaliland. The findings shed light on the mental health service needs of the student population. Subsidizing university education may be an important milestone to improve the mental health of University students as well as to ensure better access to tertiary education for the most disadvantaged communities in Somaliland. The lack of access to international scholarships for those who cannot pay tuition fees within Somaliland might also contribute to migration of the youth to other countries.

## Conclusion

A significant proportion of the students at the University of Hargeisa suffer from mental distress which might have a detrimental effect on their academic performance. The mental health needs of the University students need to be addressed with special attention paid to female students, students experiencing financial hardships, students who use khat and to those who have interpersonal problems.

## Abbreviations

AOR: adjusted odds ratio
OR: odds ratio
SRQ-20: Self Reporting Questionnaire-20
USD: United States dollars
